# Automated diagnosis of plus disease in retinopathy of prematurity using quantification of vessels characteristics

**DOI:** 10.1038/s41598-024-57072-4

**Published:** 2024-03-16

**Authors:** Sayed Mehran Sharafi, Nazanin Ebrahimiadib, Ramak Roohipourmoallai, Afsar Dastjani Farahani, Marjan Imani Fooladi, Elias Khalili Pour

**Affiliations:** 1 Retinopathy of Prematurity Department, Retina Ward, Farabi Eye Hospital, Tehran University of Medical Sciences, South Kargar Street, Qazvin Square, Tehran, Iran; 2 Ophthalmology Department, College of Medicine, University of Florida, Gainesville, FL USA; 3 Department of Ophthalmology, Morsani College of Medicine, University of South Florida, Tempa, FL USA; 4 Clinical Pediatric Ophthalmology Department, UPMC, Children’s Hospital of Pittsburgh, Pittsburgh, PA USA

**Keywords:** Eye diseases, Retinopathy of prematurity

## Abstract

The condition known as Plus disease is distinguished by atypical alterations in the retinal vasculature of neonates born prematurely. It has been demonstrated that the diagnosis of Plus disease is subjective and qualitative in nature. The utilization of quantitative methods and computer-based image analysis to enhance the objectivity of Plus disease diagnosis has been extensively established in the literature. This study presents the development of a computer-based image analysis method aimed at automatically distinguishing Plus images from non-Plus images. The proposed methodology conducts a quantitative analysis of the vascular characteristics linked to Plus disease, thereby aiding physicians in making informed judgments. A collection of 76 posterior retinal images from a diverse group of infants who underwent screening for Retinopathy of Prematurity (ROP) was obtained. A reference standard diagnosis was established as the majority of the labeling performed by three experts in ROP during two separate sessions. The process of segmenting retinal vessels was carried out using a semi-automatic methodology. Computer algorithms were developed to compute the tortuosity, dilation, and density of vessels in various retinal regions as potential discriminative characteristics. A classifier was provided with a set of selected features in order to distinguish between Plus images and non-Plus images. This study included 76 infants (49 [64.5%] boys) with mean birth weight of 1305 ± 427 g and mean gestational age of 29.3 ± 3 weeks. The average level of agreement among experts for the diagnosis of plus disease was found to be 79% with a standard deviation of 5.3%. In terms of intra-expert agreement, the average was 85% with a standard deviation of 3%. Furthermore, the average tortuosity of the five most tortuous vessels was significantly higher in Plus images compared to non-Plus images (*p* ≤ 0.0001). The curvature values based on points were found to be significantly higher in Plus images compared to non-Plus images (*p* ≤ 0.0001). The maximum diameter of vessels within a region extending 5-disc diameters away from the border of the optic disc (referred to as 5DD) exhibited a statistically significant increase in Plus images compared to non-Plus images (*p* ≤ 0.0001). The density of vessels in Plus images was found to be significantly higher compared to non-Plus images (*p* ≤ 0.0001). The classifier's accuracy in distinguishing between Plus and non-Plus images, as determined through tenfold cross-validation, was found to be 0.86 ± 0.01. This accuracy was observed to be higher than the diagnostic accuracy of one out of three experts when compared to the reference standard. The implemented algorithm in the current study demonstrated a commendable level of accuracy in detecting Plus disease in cases of retinopathy of prematurity, exhibiting comparable performance to that of expert diagnoses. By engaging in an objective analysis of the characteristics of vessels, there exists the possibility of conducting a quantitative assessment of the disease progression's features. The utilization of this automated system has the potential to enhance physicians' ability to diagnose Plus disease, thereby offering valuable contributions to the management of ROP through the integration of traditional ophthalmoscopy and image-based telemedicine methodologies.

## Introduction

Retinopathy of Prematurity (ROP) is a pathological condition characterized by the abnormal proliferation of retinal blood vessels in infants with low birth weight. This condition has significant implications for visual impairment, and if not promptly addressed, it can progress to retinal detachment and subsequent blindness^1^. ROP remains a significant and avoidable contributor to the occurrence of blindness and visual impairment among children, in both developing and developed nations^2^. It is estimated that a total of 1.84 million children were at risk of developing ROP at various stages. Among these cases, approximately 11% were expected to experience complete blindness or severe visual impairment as a direct result of ROP^3^. The prevalence of ROP in developed nations is estimated to be approximately 9%, while in developing nations, it is estimated to be around 12%^4^. In low and middle-income countries, the prevalence of ROP-induced blindness can reach up to 44%, primarily attributed to challenges related to retinal examination, patient follow-up, and the accessibility of neonatal intensive care units^5^.

The term “Plus disease” is used to characterize the most severe vascular alterations observed in ROP, which are characterized by a specific degree of vascular dilation and tortuosity.

### Screening and treatment

Multiple studies^6–8^ have demonstrated that ROP, specifically when characterized by Plus disease, can be successfully managed through laser photocoagulation^6,7^ or intravitreal injection of bevacizumab^8^ when diagnosed promptly.

Wide-field retinal imaging, such as the RetCam^9^, offers the potential to conduct tele-ROP screening in conjunction with a reading center^10,11^. This approach enhanced both the availability of ROP screening and the impartiality in diagnostic procedures. However, the clinical diagnosis of ROP continues to be subjective, resulting in significant variability and inconsistency in diagnosis. This inconsistency has been observed even among experts in ROP, as documented in previous studies^12,13^.

Several research groups have investigated the development of artificial intelligence and computer-based image analysis techniques for the purpose of improving the objectivity and automating the diagnosis of ROP with Plus disease^11,14–23^. Nevertheless, little work to our knowledge has done on quantitative analysis of image features that are focus of the clinicians during diagnosis and treatments. This study aimed to develop a computer-assisted system for detecting Plus disease by characterization of vascular features, with the intention of enhancing diagnostic accuracy and enabling quantitative monitoring of treatment progress.

## Methods

### Ethics

Five ROP experts, conducted the collection of the images and three of them participated in the labeling of fundus images. The present study adhered to the principles outlined in the Declaration of Helsinki and received approval from the institutional review board of Tehran University of Medical Sciences. Written informed consent was obtained from the parents of all patients involved in the study, granting permission for imaging and participation in the research. Furthermore, prior to the viewing of images, we took the precautionary measure of removing all sensitive information pertaining to the patients. This was done to guarantee the preservation of their anonymity and confidentiality.

### Subjects and reference standard diagnosis

We compiled a database of 76 wide-angle posterior retinal images, each of which, relates to different individual preterm infants acquired during routine clinical care. Participants in the study were 27 female (35.5%) and 49 male (64.5%) infants with an average birth weight of 1305 g (SD = 427 g) and an average gestational age of 29.3 weeks (SD = 3 weeks). The infants were examined in a participating neonatal care unit at Farabi Hospital in Tehran, Iran, between January 1, 2019 and December 30, 2020. The criteria for screening of ROP were established based on a published guideline. These criteria include a birth weight (BW) below 2000g, a gestational age (GA) of 32 weeks or less, or a determination of ROP risk by the pediatrician or neonatologist^24^. We excluded all examinations performed on eyes that had undergone prior treatment for ROP and any relevant medical conditions, such as respiratory distress syndrome, bronchopulmonary dysplasia or any systemic or ocular disease that could affect the retinal examination. Using a wide-angle imaging device (RetCam; Clarity Medical Systems, Pleasanton, CA), all images were captured. The image has a resolution of 1200 by 1600 pixels. Three ROP expert observers independently categorized selected images as “Plus” or “non-Plus”. A reference standard diagnosis (i.e., Plus or non-Plus) was defined for each of the images as the diagnosis provided by the majority of the three experts. In order to assess inter-expert and intra-expert reliability, each of the three experts submitted their labeling twice over the course of 10 days. The final dataset for this study was intended to contain 76 images, consisting of 37 non-Plus images and 39 Plus images based on the reference standard.

### Image selection and preprocessing

The challenges related to capturing retinal images of infant’s stem from inadequate patient cooperation and their limited attention span during the image acquisition process. These factors contribute to the production of low-quality images characterized by artifacts such as focusing issues, contrast deficiencies, motion blurring, and uneven illumination. Regions with low contrast and/or out of focus areas that lack distinct vessel boundaries have a detrimental impact on the segmentation of vessels and the precision of subsequent analysis. In order to address the concerns regarding image quality, an expert systematically eliminated low quality images from the dataset. In order to obtain a more accurate vessel segmentation, the selected images underwent image enhancements to address issues related to uneven illumination and to enhance the contrast between the vessels and the background retina.

This study focuses on the development of algorithms for the characterization of two main vascular image features related to Plus disease, namely the tortuosity and dilation of vessels. The performance of these algorithms in distinguishing between subjects with Plus disease and those without Plus disease was evaluated. Additionally, we conducted a comparison of the density of vessels in the retinal images to determine if there are any statistically significant differences between the two groups of subjects in relation to this particular characteristic of the vessels. The performance of our algorithms was evaluated through cross-validation using a reference standard. The reference standard was determined by aggregating the diagnoses provided by a panel consisting of three experts in ROP. In brief, the objectives of our study encompass two main aspects. Firstly, we aim to evaluate the variability in diagnosing Plus disease among different experts as well as within individual experts. Secondly, we seek to develop a computer-assisted system for detecting Plus disease, with the intention of enhancing diagnostic accuracy and enabling quantitative monitoring of treatment progress.

### Vessels segmentation

Accurate segmentation of vessels is essential for quantifying their characteristics, such as tortuosity, through extracting vessels masks. The majority of existing studies have relied on manual creation of vessels' masks for either the entire retinal image or specific vessel segments. This process involves the use of graphical editing software, such as Photoshop, which is both time-consuming and heavily reliant on the operator's skills and expertise.

In this research, the authors employed a combination of the top-hat transform technique proposed by Sharafi et al.^25,26^ for retinal vessel segmentation, along with a method introduced by Strisciuglio et al.^27^ that utilizes a set of B-COSFIRE filters specifically designed for vessel detection. By integrating these two approaches, the researchers successfully generated a vessel mask for each ROP image in an automated manner. In light of the aforementioned artifacts observed in ROP images and the imperative need for precise segmentation of vessels for subsequent analysis, we have devised a graphical user interface (GUI) aimed at rectifying the inaccuracies present in the masks generated through automated segmentation. (supplementary file-1) The segmentation of optic discs was also performed using the mentioned GUI. Our team is currently finalizing the development of a comprehensive approach that combines various methods to achieve a vessel segmentation pipeline for ROP images. This pipeline aims to achieve both high accuracy and full automation. The segmentation of vessels in ROP images was thoroughly reviewed by a ROP expert (EKP) in this study.

### Quantifying vessels’ tortuosity

Arterial tortuosity plays a crucial role in the international classification system for ROP as it is utilized in the diagnosis of Plus disease^28^. There are several studies in the literature to quantify the tortuosity of vessels as a primary characteristic for distinguishing between Plus and non-Plus^17,19,22,29^.

The utilization of distance-based metrics to quantify the tortuosity of vessels simply involves the computation of the path length traversed by a vessel segment or curve, which is then divided by the distance between the two endpoints of the vessel segment. This methodology is alternatively referred to as the Arc-over-Chord ratio which is also commonly denoted as the tortuosity index. Nevertheless, the existing body of literature presents alternative approaches for calculating tortuosity that rely on curvature, which yield more precise results. The study recently published demonstrates that curvature-based methods exhibit greater consistency and intuitiveness compared to the tortuosity index. Additionally, it highlights an inherent limitation in metrics like the tortuosity index, which fail to account for the entirety of the geometry^30^.

To achieve a desired level of accuracy, we employed the squared-derivative-curvature method^31^, which has been widely utilized in previous studies to calculate the tortuosity of retinal vessels. The tortuosity is estimated by evaluating the integral of the square of the derivative of curvature, divided by the length of the arc of the vessel segment (Eq. 1). In this particular instance, the tortuosity of a perfectly linear segment of a vessel is approximated to be 0.

1 $$ T = \frac{{\mathop \smallint \nolimits_{{t_{0} }}^{{t_{n} }} \left( {\kappa ^\prime \left( t \right)} \right)^{2} }}{{L_{c} }} $$

*κ* is the curvature of the vessel segment. In order to determine the curvature of a vessel segment, the centerline of the vessel was obtained by applying morphological thinning to the vessel segment (skeletonizing). This process involved iteratively removing pixels from the vessel's boundary. This operation maintains the topological properties and does not alter the fundamental structure of the vessel segment. Following the process of skeletonization, any spur edges that may have been present as small branches alongside the skeletonized vessel segment were effectively removed, resulting in the vessel segment having only two endpoints.

Curvature, denoted as *κ*, refers to the degree to which a curve deviates from a straight line. Curvature is related to the tangent vector at each point on a curve, as will be discussed in more detail. From a geometric standpoint, the curvature *κ* can be precisely characterized as the rate at which the tangent vector to a curve alters its orientation. In order to determine *κ*, it is necessary to first represent the skeletonized vessel segment using a continuous curve. In order to accomplish this, we estimated the skeletonized vessel segment *S* in our digital image using a cubic spline (a continuously differentiable function defined by piecewise polynomials). If *S* is represented by center line pixels located at coordinates (*x*_1_, *y*_1_), (*x*_2_, *y*_2_), …, (*x*_*n*_, *y*_*n*_) the spline is actually an interpolation over those coordinates. Nevertheless, when employing all the coordinates in set *S* for the purpose of interpolation, the resultant spline exhibits excessive noise and lacks the desired level of smoothness required to accurately represent a segment of a vessel. Consequently, it was necessary to down-sample the initial coordinates and approximate a more continuous spline function over the sampled data points.

Assuming that a particle is moving along the spline γ, then both *x* and *y* are considered as a function of a time variable, *t*, and the spline *γ* is described as γ(t) = (*x*(*t*), *y*(*t*)), where γ(t) represents the position of the particle at time *t*. The curvature *κ* at point *t,* i.e. *κ* (*t*), can be estimated by Eq. 2, where primes refer to derivatives $d/dt$ with respect to the parameter *t*.

2 $$ \kappa \left( t \right) = \frac{{\left| {x^\prime \left( t \right)y^{\prime \prime} \left( t \right){\text{ - y}}^\prime \left( t \right){\text{x}}^{\prime \prime} \left( t \right)} \right|}}{{\left( {x^\prime \left( t \right)^{2} - y^\prime \left( t \right)^{2} } \right)^{\frac{3}{2}} }} $$

*L*_*c*_, the curve length, is calculated by the formula given in Eq. 3.

3 $$ L_{c} = \mathop \smallint \limits_{{t_{0} }}^{{t_{n} }} \sqrt {1 + \left( {\frac{y^\prime \left( t \right)}{{x^\prime \left( t \right)}}} \right)^{2} } $$

**Figure 1:**
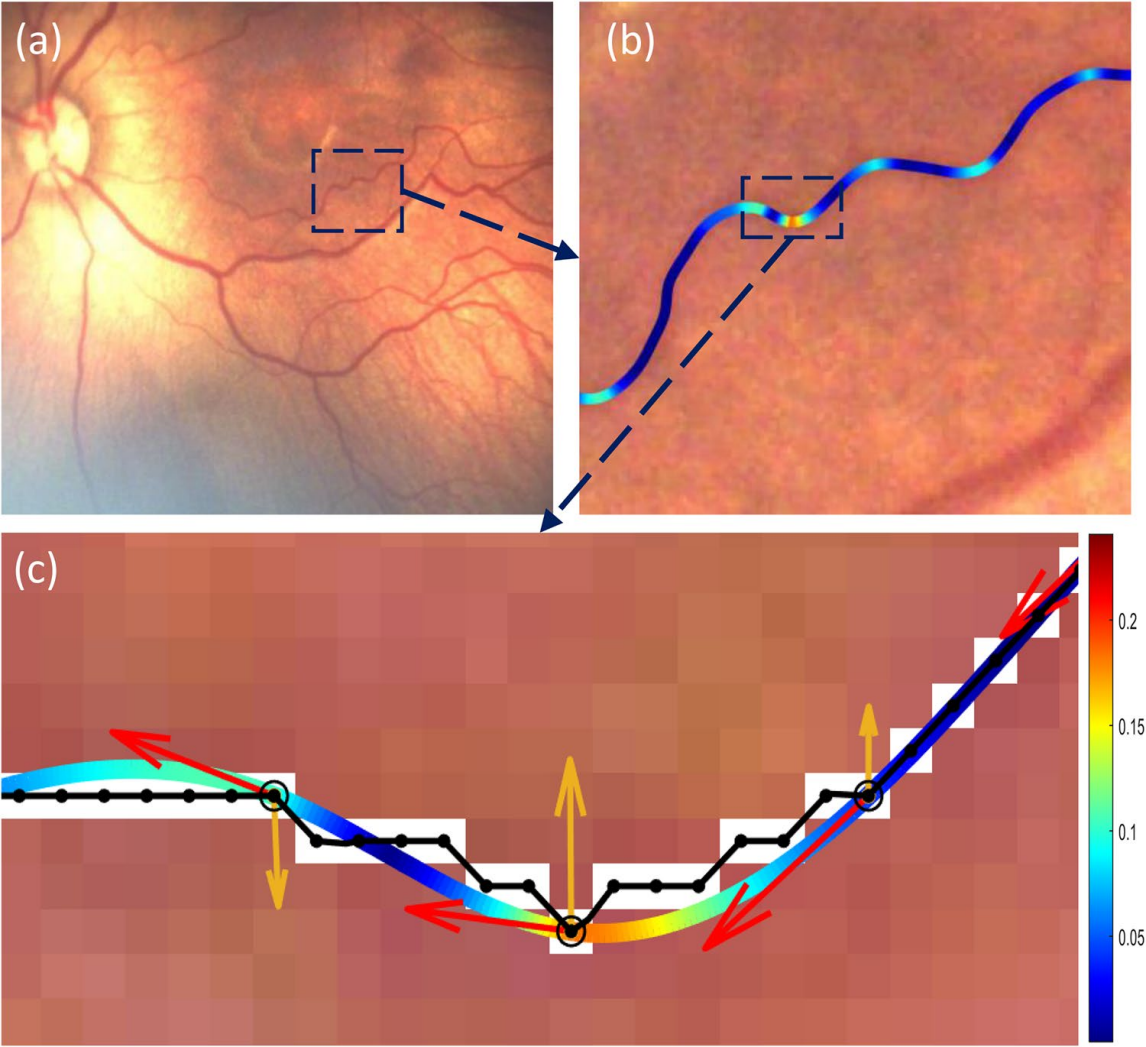
(**a**) Part of a retinal image and a single arteriole segment depicted on it (**b**) The curvature values along the vessel segment are visually represented through a color-coding scheme, where regions with high curvature are depicted in red and regions with low curvature are depicted in blue. (**c**) Centerline pixels, splines, first and second derivative at selected points: ‘.’ Shows the segment’s centerline pixels; ‘o’ shows the points after down-sampling; Black lines depict the first spline fit that passes through all the original pixels; Color-coded curve: spline fit after down-sampling and the curvature values along the spline that are represented through a color-coding scheme; Red arrows illustrate the first derivative of the spline at the selected points i.e. tangent vectors; Yellow arrows: second derivatives of the spline at the selected points i.e. acceleration vectors. Color bar shows the color map of the curvature values.

Figure 1 illustrates the sequential procedures employed for the computation of tortuosity in an arteriole segment within a retinal image obtained from a patient exhibiting Plus disease. The curvature values along the vessel segment are visually represented through a color-coding scheme, where regions with high curvature are depicted in red and regions with low curvature are depicted in blue. The first and second devivatives of the final spline at selected points are also shown as red and yellow arrows respectively.

### Vessel dilation

As previously stated, the expansion of blood vessels, known as vascular dilation, is a characteristic feature of Plus disease. To determine the diameter of each vessel segment, we employed the methodology outlined by Sharafi et al.^25^. A random selection was made of one third of the centerline pixels within the vessel segment. For each selected pixel, the shortest line connecting it to the border of the vessel segment was determined and considered as the diameter of the vessel segment at that particular pixel. The diameter of the entire vessel segment was determined by taking the average of the diameter values at the centerline pixels that were selected. The diameter index at a specific zone was defined as the maximum diameter among all vessel segments within that zone, assuming that the venule has the highest diameter in that zone. Vessel dilation measurements were performed for entire field of view and at a distance of 5 disc diameters away from the optic disc, as previously documented in the previous studies^17,20^.

### Vessel density

Since ROP is a proliferative retinal vascular disease and Plus disease is characterized by vascular tortuosity and dilation, it is hypothesized that the vessel density in Plus disease is greater than that of a healthy retina. The density of retinal vessels has been extensively discussed in the academic literature as a potential indicator of ROP^32–36^, particularly in relation to Plus disease^35^. Therefore, in our study, we investigated the density of vessels as a potential indicator of Plus disease, in addition to evaluating tortuosity and dilation. The calculation of vessel density involved determining the ratio of the number of pixels located on the vessels of each image to the number of pixels located on the remaining portion of the entire vascularized region. The vascularized region was acquired through the application of morphological image dilations on the vessel mask.

## Results

### Inter-expert and intra-expert variability and the expert’s agreement

Cohen's kappa statistics were computed to evaluate the level of agreement between experts and the consistency of grades assigned by a single expert across two separate sessions. Tables 1 and 2 present the levels of inter-expert and intra-expert variability, respectively. Table 3 presents the concordance between individual experts and the reference standard. The average level of agreement among experts was found to be 79% with a standard deviation of 5.3%. In terms of intra-expert agreement, the average was 85% with a standard deviation of 3%.

**Table 1:** Inter-expert agreement.

Expert pairs	Kappa	Absolute agreement (%)
E1E2	0.7105	85.52
E1E3	0.5816	78.94
E2E3	0.5000	75.00

**Table 2:** Intra-expert agreements in two different sessions.

Experts	Kappa	Absolute agreement (%)
E1E1	0.6344	81.58
E2E2	0.7368	86.84
E3E3	0.7057	86.84

**Table 3:** Agreement for individual experts with the reference standard.

Expert vs. reference standard	Kappa	Absolute agreement (%)
E1R	0.8948	94.74
E2R	0.8158	90.79
E3R	0.6822	84.21

### Feature extraction and features impact

**Figure 2:**
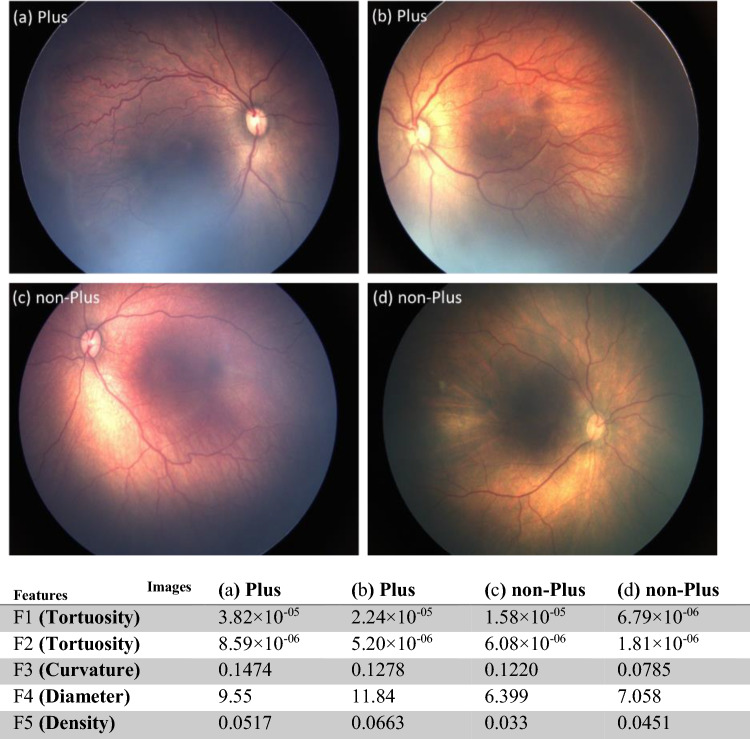
Two samples of Plus images (**a** and **b**) and two samples of non-Plus images (**c** and **d**) and their corresponding feature values.

In accordance with the preceding section, a total of 10 features were initially extracted to delineate the attributes of tortuosity, dilation, and vessel density. In order to prioritize more discerning characteristics, we employed the Neighborhood Component Analysis (NCA) technique for feature selection^37^. Ultimately, we identified and selected five features with greater weights. The selected features and their attributes are presented in Table 4. To represent some sample images of our data set and the corresponding features’ values calculated for each of the samples, Fig. 2 shows two samples of Plus images and two sample of non-Plus images and their associated feature values.

**Table 4:** List of the selected features, description, region of the image that the feature calculated for, and the method used to calculate the feature.

Feature category	Feature name	Description	Region	Calculation method	*p* value (Bonferroni -corrected)
Tortuosity	F1	Average values of the 5 most tortuous segments	Entire image	Equation 1	< 0.0001
	F2	Average tortuosity of all segments	5DD	Equation 1	< 0.0001
	F3	Average of top 1% curvature values	Entire image	Equation 2	< 0.0001
Diameter	F4	Diameter of the thickest segment	5DD	Average diameter at random points along each of the vessel segments	< 0.0001
Density	F5	Ratio of the area occupied by the vessels to the rest of the vascularized area	Entire image	Vascularized area determined by morphological dilation applied on the vessel mask	< 0.0001

**Figure 3:**
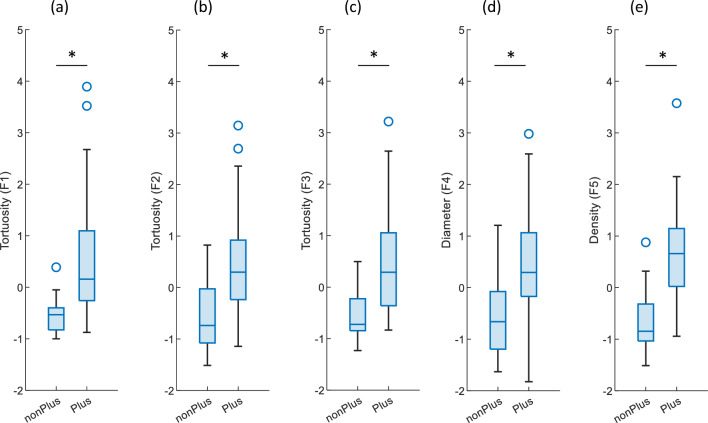
Comparison of extracted feature values between Plus and non-Plus images (values are standardized). (**a**) Mean tortuosity of the five vessel segments with the greatest tortuosity (F1). (**b**) The mean tortuosity of all vessel segments within a region extending 5 diameters from the OD border (5DD) (F2). (**c**) The mean of the highest 1% of curvature values (F3). (**d**) The maximum diameter of the vessel in the 5DD region (F4). (**e**) Vessel density determined in the retina's vascular region (F5). Blue circles represent outlier data values.

Figure 3 presents a comparison between Plus and non-Plus images with respect to the selected features outlined in Table 4. Figure 3a presents a comparison between Plus and non-Plus images in terms of the average tortuosity values observed in five vessel segments that exhibit the highest levels of tortuosity within the entire image (F1). There was a significant difference observed between the measure of Plus images and non-Plus images (*p* ≤ 0.0001). Figure 3b presents a comparison between Plus and non-Plus images in terms of the average tortuosity of their vessel segments within a region located 5 diameters away from the optic disc border, referred to as 5DD (F2). The tortuosity measure of Plus images exhibited a statistically significant difference compared to the non-Plus images (*p* ≤ 0.0001). Figure 3c presents a comparative analysis between Plus images and non-Plus images in relation to feature F3, specifically focusing on the average of the top 1% curvature values. There was a significant difference observed between the measure of Plus images and non-Plus images (*p* ≤ 0.0001). Figure 3d presents a comparison between Plus and non-Plus images in terms of the maximum vessel diameter observed within the 5DD region (F4). The diameter measurements of Plus images exhibited a significantly greater magnitude compared to non-Plus images (*p* ≤ 0.0001). Figure 3e presents a comparison of vessel density between Plus images and non-Plus images (F5). The density of vessels in Plus images was significantly greater than that in non-Plus images (*p* ≤ 0.0001).

### Image classification

**Figure 4:**
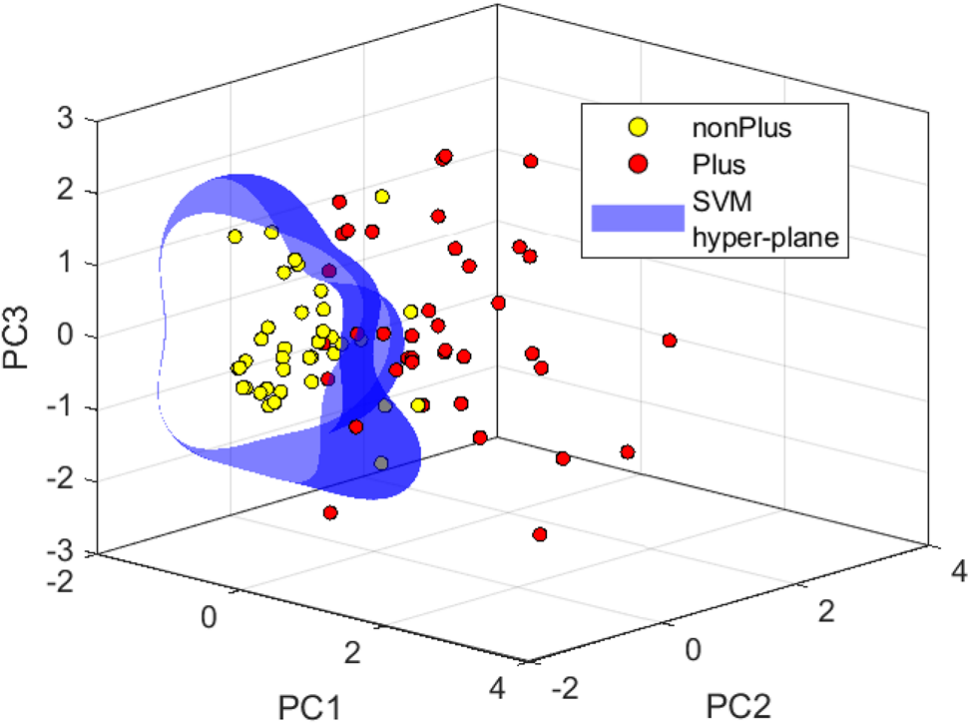
Datapoints associated with Plus and non-Plus images depicted by the first, second, and the third principal components of the selected features (values are standardized).

Since three out of the five selected features (i.e. F1, F2, and F3) were in the category of the vessels’ tortuosity and they were likely to be correlated, we tried for more dimension reduction by utilizing Principal Component Analysis (PCA) on the selected features. We yielded the best accuracy with the first, second, and third components and using them to train an SVM classifier. A radial basis kernel function and the regularization parameter of 1 were used for the SVM classification. The performance of our classifier was assessed using a tenfold cross validation. We also assessed the accuracy of our classifier in comparison to the accuracy of the diagnosis provided by experts for the reference standard. Table 5 displays the diagnostic accuracy of each expert and the proposed method in distinguishing Plus images from non-plus images. According to the table, the proposed method achieved an accuracy of 0.86 ± 0.01, placing it third among the accuracy values of the expert-provided diagnosis. The accuracy values for our classifier were obtained through repetitive execution of tenfold cross-validation. Figure 4 illustrates the data points of Plus and non-Plus images in a three-dimensional plane, utilizing the standardized values of the first, second, and third principal components of the chosen features.

**Table 5:** Comparison of the accuracy measures of the expert-provided diagnosis and the proposed method's prediction against the reference standard.

Experts/algorithm prediction (ordered by accuracy)	Accuracy	Sensitivity	Specificity	PPV	NPV
E1	0.94	0.92	0.97	0.97	0.92
E2	0.90	0.89	0.91	0.9210	0.89
Proposed algorithm	0.86 ± 0.01	0.89 ± 0.02	0.83 ± 0.01	0.85 ± 0.02	0.88 ± 0.02
E3	0.84	0.94	0.72	0.78	0.93

**Figure 5:**
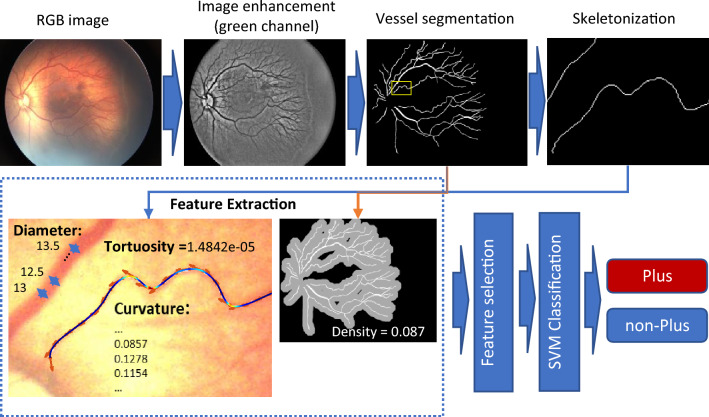
A Schematic of the proposed method and sample outputs at each stage.

Figure 5 illustrates an overall view of the proposed method and a schematic of the outputs at each of the stages.

## Discussion

Numerous research groups have undertaken investigations into the advancement of computer-based image analysis techniques for the purpose of automating the diagnosis of ROP with Plus disease^11,14–18,38–42^.

In order to establish an automated system for the detection of ROP, it is necessary to conduct an analysis of retinal fundus images and accurately characterize the distinctive features associated with ROP. The identification of ROP and more specifically, the presence of Plus disease, can be conceptualized as a classification task within the domain of machine learning. Conventional algorithms employ handcrafted features (HC), such as vessel dilation and tortuosity, to analyze retinal fundus images and distinguish between Plus disease and pre-Plus/non-Plus conditions^17,19,20,43,44^. In contrast, Deep Convolutional Neural Networks (DCNN) possess the ability to acquire knowledge of image features from the provided inputs in order to effectively classify labels. DCNNs have demonstrated effective utilization in the automated identification of various ocular diseases, including diabetic retinopathy, glaucoma, age-related macular degeneration, and retinopathy of prematurity. These findings have been reported in multiple studies^14,18,21,45–48^. DCNN often requires a substantial quantity of high-quality training samples that are well-balanced across different labels. However, obtaining such samples in the context of ROP can be challenging due to limited patient cooperation and inadequate attention span, particularly in children. Additionally, the features acquired by DCNNs lack transparency and interpretability. Therefore, the utilization of HC features in the detection of Plus Disease has proven to be effective, both as a standalone method^17,22,29^ and as a means of providing supplementary information for DCNN in image classification tasks^16,23^.

In addition to the diagnosis of Plus disease, a significant challenge encountered by experts in the field of ROP pertains to the assessment of the efficacy of clinical interventions implemented during patient treatments^49–51^. The quantification of vascular characteristics during therapeutic procedures can provide clinicians with a more precise means of monitoring the effects of their interventions on the disease.

Accurate vessel segmentation is crucial for quantifying vessel characteristics, such as tortuosity, by extracting vessel masks. Most studies have manually created masks for vessels in retinal images, either for the entire image or for specific segments of vessels. This process utilizes graphical editing software, such as Photoshop, which requires significant time and relies heavily on the operator's proficiency and expertise. In the current study we utilized a combination of thresholding technique for retinal vessel segmentation, along with a method utilizes a set of B-COSFIRE filters designed for vessel detection^27^. By integrating these two approaches, the researchers achieved automated generation of a vessel mask for each ROP fundus image. We built a GUI to correct automated segmentation mask inadequacies due to ROP image artifacts and the need for proper vessel segmentation for subsequent analysis. Accurate and automated vessel segmentation in ROP fundus images can also serve as a dataset for a comprehensive vessel segmentation method using deep learning in our future studies.

This study aimed to develop algorithms for characterizing two primary vascular image features associated with Plus disease: vessel tortuosity and dilation. The algorithms were assessed for their ability to differentiate between subjects with Plus disease and those without Plus disease. It was demonstrated that the mean tortuosity of the five most tortuous vessels exhibited a statistically significant increase in Plus images when compared to non-Plus images (*p* ≤ 0.0001). The analysis revealed that the curvature values derived from points exhibited a statistically significant increase in Plus images as compared to non-Plus images (*p* ≤ 0.0001). A statistically significant increase in the greatest diameter of vessels within a zone extending 5-disc diameters away from the edge of the optic disc (referred to as 5DD) was observed in Plus images compared to non-Plus images (*p* ≤ 0.0001). In addition to vessel tortuosity and dilation, our method validates the finding by Ataer et al. 26 that the point-based curvature values are substantially greater in Plus images than in non-Plus images.

Additionally, we conducted a comparative analysis of vessel density in retinal images to evaluate any statistically significant differences between the two subject groups in relation to this particular characteristic of blood vessels. The density of vessels in Plus images was significantly higher than in non-Plus images (*p* ≤ 0.0001).

The literature extensively discusses the density of retinal vessels as a potential indicator of ROP, particularly Plus disease. The relationship between vessel density and Plus disease remains unclear, as there is limited research on this specific aspect. Additionally, the development of Plus disease is influenced by multiple factors, including gestational age, birth weight, and the overall health of the infant. The study findings revealed a significant difference in vessel density between Plus and non-Plus images (*p* ≤ 0.0001). This finding supports the results of a study conducted by Mao et al.^35^, which demonstrated that patients diagnosed with Plus and pre-Plus exhibited significantly higher vessel density compared to the normal group. They also demonstrated a proportional decrease in vessel density at 7-, 14-, and 30-days post-treatment. An increase in vessel density was observed in a study conducted on a mouse model of oxygen-induced retinopathy (OIR)^32^. However, our findings contradict other studies that did not observe a significant increase in vessel density between the ROP group and the normal group^33,34^.

We trained an SVM classifier using the extracted features to distinguish between Plus and non-Plus images. We evaluated the performance of our algorithms using cross-validation and a reference standard. The reference standard was determined through the integration of the diagnoses given by a panel consisting of three experts in ROP. The classifier's accuracy in distinguishing between Plus and non-Plus images, as determined through tenfold cross-validation, was 0.86 ± 0.01. The observed accuracy was higher than that one of the three experts in comparison to the reference standard.

There is considerable variation in the classification of Plus disease by experts in ROP. This variation arises from differences in the thresholds used by experts to determine the extent of vascular abnormality necessary to diagnose Plus and pre-Plus disease. This finding has significant implications for ROP studies, instruction, and patient care. It indicates that a continuous ROP Plus disease severity score could provide a more accurate representation of expert ROP clinicians' assessments and potentially improve the standardization of classification in the future^52^.

The Images and Informatics in ROP(i-ROP) deep learning (DL) algorithm (i-ROP) is a well-known AI tool used to measure vascular changes in the posterior pole in cases of ROP. Multiple studies on ROP screening have shown that the i-ROP algorithm can effectively detect Plus disease, similar to human ROP experts. This suggests that the algorithm has the potential to identify cases of ROP reactivation that require retreatment. The i-ROP DL system's output can be converted into a vascular severity score (VSS) that represents the range of Plus disease. This score has proven to be valuable for primary ROP screening, tracking disease progression, and evaluating treatment response^15,16,49,50,53–56^.

The study demonstrated the utility of VSS in both primary and secondary ROP screening, including the screening for ROP reactivation following anti-VEGF treatment^51^.

Infants diagnosed with ROP in developing countries tend to have higher birth weights and older gestational ages compared to those in developed countries. As a result, broader screening guidelines have been implemented in low- and middle-income countries, leading to a larger population at risk for ROP in these regions^57^. The rise in screening burden poses a particular challenge due to the relatively lower number of ophthalmologists per capita in comparison to higher-income nations. The current study, aimed to develop a computer-assisted system for detecting Plus disease in Iran as a low-income country, with the goal of improving diagnostic accuracy and facilitating quantitative monitoring of treatment progress. This study quantified the characteristics of vessels in ROP images, focusing on tortuosity, dilation, and density. These measurements were used for primary Plus disease screening and could be useful for future studies on screening for ROP reactivation after anti-VEGF treatment.

This study has a number of limitations. Firstly, the dataset used in this study had a limited number of ROP fundus images, which may have influenced the performance of the model. Secondly, the fundus images were collected from a single clinical site with consistent device settings and population characteristics, which could have reduced the diversity of the data and affected the algorithm's ability to generalize to other populations. Lastly, while a cross-validation method was employed to enhance generalizability, further validation using independent images is necessary for future research.

Future studies should aim to obtain larger datasets of ROP images in order to validate and optimize our system within the clinical setting. Additionally, it may be necessary to conduct additional testing and optimization of the sensitivity metric in order to minimize the occurrence of false-negative results. Additional studies are required to validate this automated system and enhance its practicality for real-world clinical applications by incorporating datasets from multiple clinical centers and larger patient cohorts.

In conclusion, the algorithm used in this study showed high accuracy in detecting Plus disease in cases of retinopathy of prematurity, performing similarly to expert diagnoses. By objectively analyzing vessel characteristics, it is possible to quantitatively assess the features of disease progression. The automated system has the potential to improve physicians' ability to diagnose Plus disease, making valuable contributions to the management of ROP by integrating traditional ophthalmoscopy and image-based telemedicine methods.

### Supplementary Information

Supplementary Figures.

## Data Availability

The datasets developed and/or analyzed during the current study are available from the corresponding author upon reasonable request.
