# Supplementary material for: Automated diagnosis of plus disease in retinopathy of prematurity using quantification of vessels characteristics
Source: Sci Rep. 2024 Mar 16;14:6375. doi: 10.1038/s41598-024-57072-4 (PMC10944526; doi:10.1038/s41598-024-57072-4)
Supplement: Supplementary file 1 — Supplementary Figures. [file 41598_2024_57072_MOESM1_ESM.pdf]

Supplementary Materials

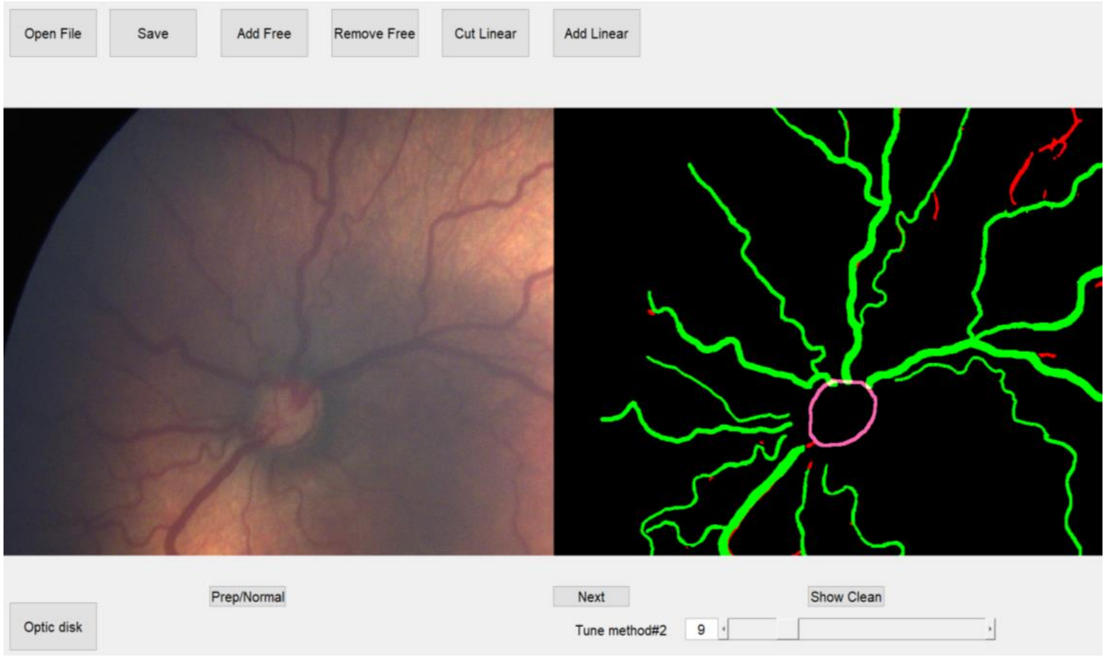

Figure A: GUI schema developed in MATLAB Software (The MathWorks, Inc.) for correcting vessels' mask after the automated segmentation.

Figure annotations:

- Left View: Original (or preprocessed) image with a synchronous zoom/pan ability with the mask appeared on the Right View.
- Right View: View of the vessel mask: green area illustrates the pixels that are agreed to be vessels by both the segmentation methods. Red area illustrates the pixels that are not agreed to be part of vessels by either of the two segmentation methods. Purple line shows the optic disk region drawn by the user. By clicking on each pixel, user can add or remove that pixel and all the connected pixels to/from the vessel mask. It also toggles the color of the clicked area between red (out of the vessel mask) and green (belongs to the vessel mask).
- Open File: Opens an ROP image file; after selecting the image file by the user, masks and current status of the workflow (added areas, removed areas etc.) are loaded and the vessel mask is shown on the Right View. If there are no previous work on the image, segmentation algorithms are executed to produce the mask for the first time.
- Save: Saves the current status of the work flow, vessel mask, and the optic disk's mask.
- Add Free: Is used to add a missed piece of a vessel to the vessel mask using a resizable pen.
- Remove Free: Removing an area from the vessel mask. The removed area is shown in the mask with red color thereafter.
- Cut Linear: Cutting a piece of a vessel mask to disconnect it from the rest of the mask.
- Add Linear: Same as Add Free but for precisely adding linear segments for very small areas.
- Prep/Normal: Switches the left side view between normal view and the preprocessed view of the image; in preprocessed view: 1) illumination deformity is corrected, and 2) contrast of the vessels and the background retina is enhanced so that the user can see tiny vessels that are hardly distinguishable in the Normal view. (Figure B, shows the preprocessed image shown in the left view of figure).
- Show Clean: Showing just the confirmed areas of the vessel mask (green pixels) as a black and white image.
- Optic disk: A facility to determine the area of the optic disk by the user.
- Tune method#2: Changes the threshold applied on the filter response of the second method of segmentation (described in Vessel Segmentation part of the Methods section) either by inputting a number or by changing the slider bar. With combination of the first method of the segmentation, increasing or decreasing this threshold, produces a mask with lower or higher false positives respectively. Normally, the user utilizes this facility at the beginning of the workflow to obtain the best possible result of the automatic segmentation before doing any manual corrections.

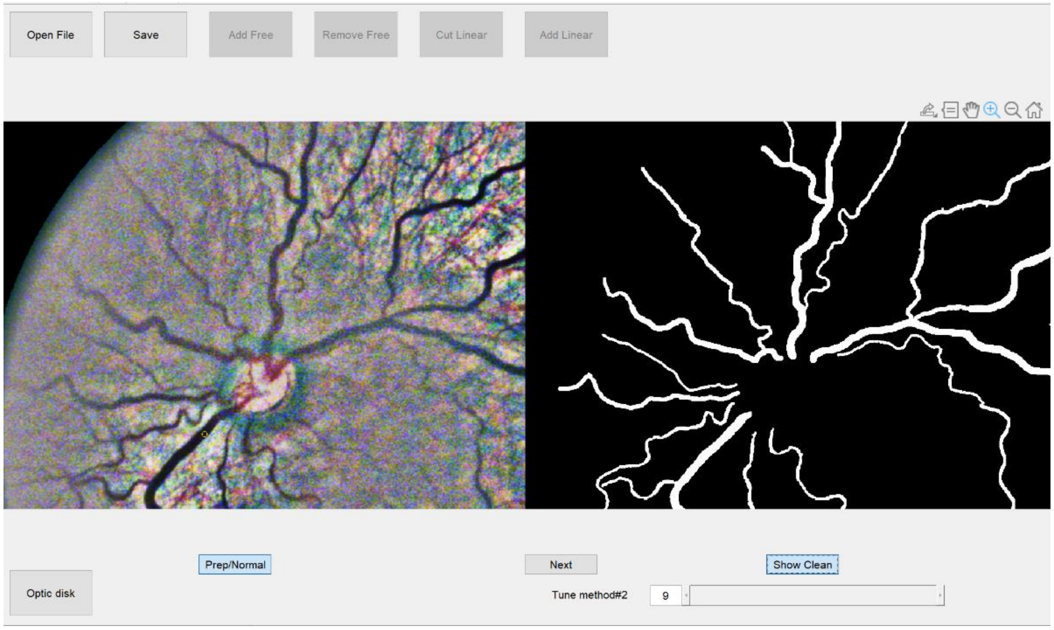

Figure B: Left view: preprocessed version of the image shown in Figure A. Right view: the vessel mask corresponding to the image at the left view that appeared after pushing the "Prep/Normal" and "Show Clean" buttons (right view).

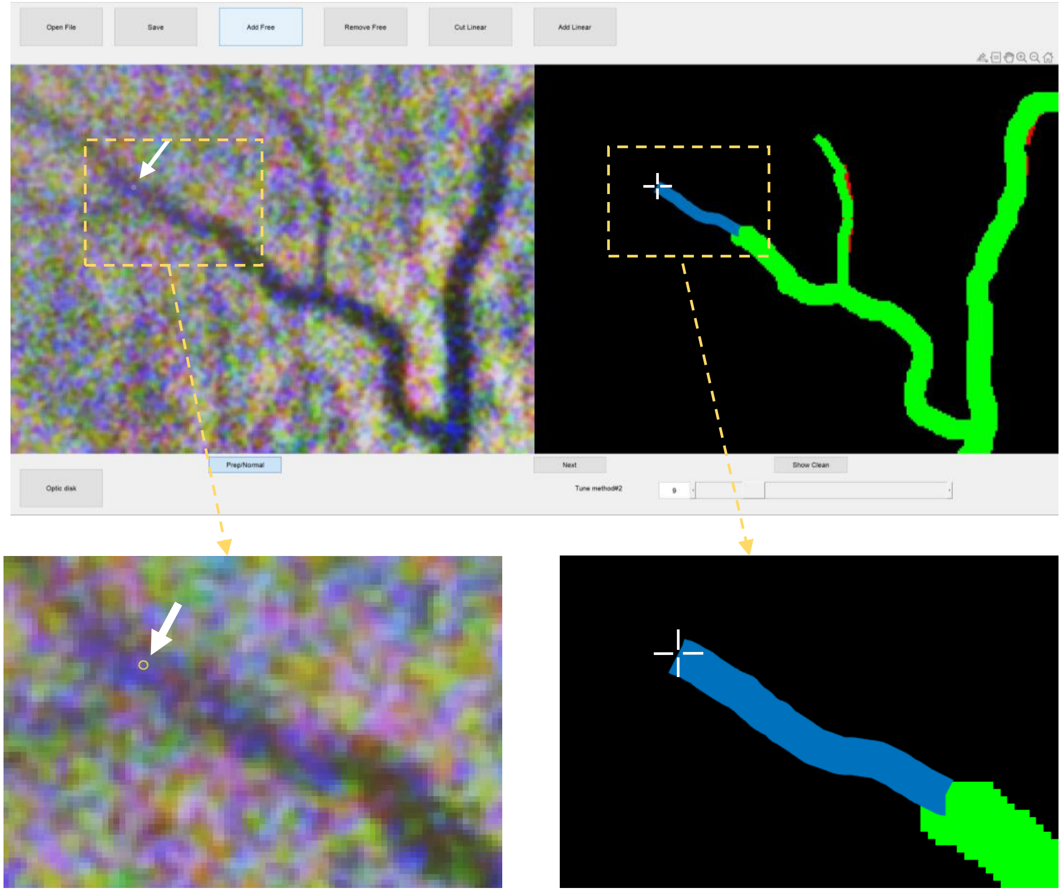

Figure C: Manual correction of a misclassified piece of a vessel by utilizing "Add Free" with a size of 3 pixels. To prevent mistakes in manual correction, cursor on the right view ('+' symbol) is always pointing to the corresponding coordinates in the left view (yellow circle depicted by white arrow) and moving synchronously.
